# Direct Comparison of [^18^F]F-DPA with [^18^F]DPA-714 and [^11^C]PBR28 for Neuroinflammation Imaging in the same Alzheimer’s Disease Model Mice and Healthy Controls

**DOI:** 10.1007/s11307-021-01646-5

**Published:** 2021-09-20

**Authors:** Francisco R. López-Picón, Thomas Keller, Diana Bocancea, Jatta S. Helin, Anna Krzyczmonik, Semi Helin, Annelaure Damont, Frédéric Dollé, Juha O. Rinne, Merja Haaparanta-Solin, Olof Solin

**Affiliations:** 1 PET Preclinical Imaging Laboratory, Turku PET Centre, University of Turku, Turku, Finland; 2 MediCity Research Laboratory, University of Turku, Turku, Finland; 3 Radiopharmaceutical Chemistry Laboratory, Turku PET Centre, University of Turku, Turku, Finland; 4 CEA, I2BM, Service Hospitalier Frédéric Joliot, Orsay, France; 5 Turku PET Centre, University of Turku, Turku, Finland; 6 Division of Clinical Neurosciences, Turku University Hospital, Turku, Finland; 7 Department of Chemistry, University of Turku, Turku, Finland; 8 Accelerator Laboratory, Turku PET Centre, Åbo Akademi University, Turku, Finland

**Keywords:** Alzheimer’s disease, Neuroinflammation, [^18^F]F-DPA, [^18^F]DPA-714, [^11^C]PBR28, TSPO, PET, Microglia

## Abstract

**Purpose:**

In this study we compared the recently developed TSPO tracer [^18^F]F-DPA, with [^18^F]DPA-714 and [^11^C]PBR28 by performing *in vivo* PET imaging on the same Alzheimer’s disease mouse model APP/PS1-21 (TG) and wild-type (WT) mice with all three radiotracers.

**Procedures:**

To compare the radiotracer uptake, percentage of injected dose/mL (%ID/mL), standardized uptake value ratios to cerebellum (SUVR_CB_), and voxel-wise analyses were performed.

**Results:**

The peak uptake of [^18^F]F-DPA was higher than 4.3% ID/mL, while [^18^F]DPA-714 reached just over 3% ID/mL, and [^11^C]PBR28 was over 4% ID/mL in only one brain region in the WT mice. The peak/60-min uptake ratios of [^18^F]F-DPA were significantly higher (*p* < 0.001) than those of [^18^F]DPA-714 and [^11^C]PBR28. The differences in [^18^F]F-DPA SUVR_CB_ between WT and TG mice were highly significant (*p* < 0.001) in the three studied time periods after injection. [^18^F]DPA-714 uptake was significantly higher in TG mice starting in the 20–40-min timeframe and increased thereafter, whereas [^11^C]PBR28 uptake became significant at 10–20 min (*p* < 0.05)**.** The voxel-wise analysis confirmed the differences between the radiotracers.

**Conclusions:**

[^18^F]F-DPA displays higher brain uptake, higher TG-to-WT SUVR_CB_ ratios, and faster clearance than [^18^F]DPA-714 and [^11^C]PBR28, and could prove useful for detecting low levels of inflammation and allow for shorter dynamic PET scans.

**Supplementary Information:**

The online version contains supplementary material available at 10.1007/s11307-021-01646-5.

## Introduction

Neuroinflammation is associated with several neurological diseases, such as multiple sclerosis (MS), Alzheimer’s disease (AD), and stroke. Innate pathology triggers inflammation that induces an increase in the expression of mitochondrial 18-kDa translocator protein (TSPO) in the microglia. TSPO is the main target for the PET tracers currently used for imaging neuroinflammation *in vivo* [1–5].

Although [^11^C]PK11195, one of the first TSPO PET tracers, is still routinely used in clinical imaging, it has several limitations, including poor signal-to-noise ratio, high lipophilicity, low blood–brain barrier penetration, and the short half-life of carbon-11 [6, 7]. Consequently, numerous second-generation PET tracers have been developed, including [^11^C]PBR28, [^18^F]GE180, [^18^F]DPA-714, and [^18^F]F-DPA, to name a few [8–12].

The half-life of fluorine-18 (t_1/2_ = 109.8 min) makes it a more desirable radioisotope than carbon-11 (t_1/2_ = 20.3 min) for the development of radiotracers because of the possibility of distribution to PET centers lacking an on-site cyclotron. Furthermore, fluorine-18 emits positrons with low energy (E_β+ max_ = 0.63 MeV). Consequently, the positrons have a short range in tissue and provide higher-resolution PET images than those afforded by carbon-11 radiotracers. For these reasons, several ^18^F-labeled tracers have been developed, notably among them [^18^F]DPA-714, which presents good binding potential and bioavailability. According to several animal studies, [^18^F]DPA-714 is better for PET imaging than [^11^C]PK11195, due to its low nonspecific binding in the brain and the longer half-life of the labeling radionuclide [13, 14]. Recent longitudinal studies using mouse and rat models of AD have shown an increase in [^18^F]DPA-714 uptake with disease progression [15–17]; however, the results of human [^18^F]DPA-714 PET studies in AD patients have been contradictory [18–20].

We have previously published the synthesis of [^18^F]F-DPA, an analogue of [^18^F]DPA-714, by an electrophilic ^18^F-labeling route and showed that the position of the label directly on the aromatic moiety imparts a higher *in vivo* stability than that of [^18^F]DPA-714 in Sprague–Dawley rats [12]. More recent studies have demonstrated the specificity of [^18^F]F-DPA towards TSPO and its usefulness in imaging glial activation in the APP-PS1/21 mouse model of AD [11] and in a model of ischemic stroke [21]. In addition, a study comparing electrophilic and nucleophilic syntheses of [^18^F]F-DPA demonstrated that a 100-fold difference in injected mass of [^18^F]F-DPA affected both the tracer kinetics and uptake in the APP-PS1/21 mouse model. The higher injected mass gave a faster washout and more rapid establishment of tracer equilibrium while still providing a significant uptake difference between age-matched TG and WT animals [10].

In this study we chose to compare [^18^F]F-DPA synthesized by the electrophilic approach with the clinically used TSPO tracers [^18^F]DPA-714 and [^11^C]PBR28 in the same TG and WT mice. The relative ease of the electrophilic synthetic procedure in our hands, the favorable kinetics of the tracer, and the high abundance of the target in TG mice motivated this study.

## Materials and Methods

### Tracer Synthesis

[^18^F]F-DPA and [^18^F]DPA-714 were synthesized according to the previously described procedures [19]. Two syntheses of [^18^F]F-DPA and [^18^F]DPA-714 were used for this study, and the final products were obtained with molar activities of 7.5 and 7.2 GBq/µmol for [^18^F]F-DPA and greater than 1 TBq/µmol for [^18^F]DPA-714. Six different [^11^C]PBR28 syntheses were produced with a mean molar activity of 570 GBq/µmol. All molar activities are decay-corrected to the start of radiosynthesis. [^11^C]PBR28 production was adapted from a published method [22], with some modifications. The detailed description of [^11^C]PBR28 production is included in the Supplementary information.

### Animals

Six female transgenic APP-PS1/21 (TG) mice (9 months old; 27 ± 2 g) and 6 female WT (9 months old; 34 ± 6 g) littermates were used for this study. APP/PS1-21 mice (C57BL/6 J–TgN(Thy1–APPKM670/671NL; Thy1–PS1L166P) [23] were originally provided by Koesler (Rottenburg, Germany). All animals were group-housed under standard conditions (temperature 21 ± 1.2 °C, humidity 55 ± 5%, with a 12-h light/dark cycle and ad libitum soy-free chow (RM3 [E] soya-free, 801,710, Special Diets Service, UK) and tap water. All animal experiments were approved by the Regional State Administrative Agency for Southern Finland (ESAVI/4499/04.10.07/2016 and ESAVI/3899/0404.10.07/2013), and the animal care complied with the guidelines of the International Council of Laboratory Animal Science (ICLAS). The study was performed in strict compliance with the ARRIVE guidelines and met the principles of the 3Rs (Replacement, Reduction, and Refinement) by using PET imaging to examine the same animals repeatedly.

### In vivo* Binding of [*^*18*^*F]F-DPA, [*^*18*^*F]DPA-714, and [*^*11*^*C]PBR28*

The same 9-month-old WT (*n* = 6) and TG (*n* = 6) mice were imaged using [^11^C]PBR28 [^18^F]DPA-714 and [^18^F]F-DPA within a period of 10 days. The mice, anesthetized with a 2.5% isoflurane/oxygen mixture 30 min prior to tracer injection, were injected via a tail vein with [^18^F]F-DPA (injected dose 6.9 ± 0.2 MBq; 43 ± 18 μg/kg), [^18^F]DPA-714 (injected dose 6.8 ± 0.3 MBq; 0.8 ± 0.3 μg/kg), or [^11^C]PBR28 (injected dose 10.3 ± 0.8 MBq; 0.4 ± 0.1 μg/kg) for 60-min dynamic scanning using an Inveon multimodality PET/computed tomography (CT) scanner (Siemens Medical Solutions, Knoxville, TN, USA). A few drops of Oftagel (2.5 mg/g; Santen, Tampere, Finland) were applied to the eyes of the animals to prevent eye dryness. The scanner has an axial 12.7-cm field of view and 10-cm transaxial field of view, generating images from 159 transaxial slices of voxel size of 0.78 × 0.78 × 0.8 mm^3^. CT preceded the PET modality for attenuation correction and anatomical reference. One of the TG animals imaged with [^18^F]F-DPA died during the scan, and hence this animal was excluded from the analysis.

### Analysis of PET Data

The PET/CT images were pre-processed in MATLAB R2017a (The MathWorks, Natick, Massachusetts, USA) with an in-house semi-automated pipeline for preclinical images that use SPM12 (Wellcome Department of Cognitive Neurology, London, UK) pre-processing functionalities and analysis routines. Images were first cropped to a bounding box containing the heads, and individual PET images were co-registered through a rigid-body transformation to their corresponding CT scan. Subjects were spatially normalized through a two-step registration (a rigid followed by an affine transformation) of each subject’s CT to a template CT that was previously constructed as an average of several subjects and was aligned with an atlas T2-weighted MRI template [24]. The combination of transformations was then applied to the PET images, which were also re-sampled to a voxel size of 0.2 × 0.2 × 0.2 mm (trilinear interpolation), matching the anatomical atlas dimensions.

Volume of interest (VOI) analysis of whole brain, cortex, hippocampus (HIPPO), striatum (STR), thalamus (THA), and cerebellum (CB) was performed on each subject by averaging the signal inside a slightly modified version of the Ma et al. [24] atlas-delineated VOIs. Data were obtained as the percentage of injected dose/mL (%ID/mL) or standardized uptake value ratios to cerebellum (SUVR_CB_).

Prior to the voxel-wise analysis, single static frames representing three time periods after tracer injection (10–20 min, 20–40 min, and 40–60 min) were constructed by averaging the corresponding frames of the dynamic scans for each subject and each tracer. The resulting static images were intensity-normalized to the CB as a reference region (tissue-to-reference ratio images) and smoothed using an isotropic Gaussian kernel of 0.5 mm full width at half maximum. Ratio images were masked using a whole brain mask to remove the extra-cerebral signal prior to the statistical parametric mapping analysis, and the analysis was performed without global normalization due to the use of ratio images. Between-group effects were tested for each tracer using a voxel-wise two-sample t-test. An uncorrected voxel-level significance threshold (*p* < 0.01) was used, and subsequently a cluster-level family-wise error correction at *p* < 0.01 was applied.

### Data Analysis and Statistics

The results are reported as average ± standard deviations (SDs). All statistical analyses were calculated using GraphPad Prism (GraphPad Software, v. 5.01, San Diego, CA, USA).

The differences in [^18^F]F-DPA, [^18^F]DPA-714, and [^11^C]PBR28 SUVR_CB_ between TG and WT animals in the different time frames shown in Fig. 2 were calculated using the Mann–Whitney test.

The differences in peak/60-min ratios shown in Table 1 for [^18^F]F-DPA, [^18^F]DPA-714, and [^11^C]PBR28 and the differences between TG/WT ratios for [^18^F]F-DPA, [^18^F]DPA-714, and [^11^C]PBR28 for the different brain regions shown in Table 2 were analyzed using repeated-measures ANOVA with Tukey’s post hoc test for multiple comparison.

**Table 1.** Averaged percentage of injected dose/mL (%ID/mL) for [^18^F]F-DPA, [^18^F]DPA-714, and [^11^C]PBR28 at peak uptake and 60 min after injection, together with peak/60 min ratios

	[^18^F]F-DPA			[^18^F]DPA-714			[^11^C]PBR28		
	Peak	60 min	Peak/60 min	Peak	60 min	Peak/60 min	Peak	60 min	Peak/60 min
Brain	4.83	1.37	3.52	2.69	1.79	1.50 ^***^	3.59	1.89	1.90^***^
Cortex	4.26	1.17	3.64	2.35	1.51	1.55 ^***^	3.13	1.60	1.95 ^***^
Hippocampus	4.84	1.13	4.27	2.94	1.68	1.75 ^***^	3.89	1.72	2.26 ^***^
Striatum	4.72	1.07	4.41	2.57	1.46	1.76 ^***^	3.64	1.59	2.29 ^***^
Thalamus	5.29	1.05	5.02	3.02	1.54	1.96 ^***^	4.10	1.63	2.52 ^***^

Averaged %ID/mL for [^18^F]F-DPA, [^18^F]DPA-714, and [^11^C]PBR28 (*n* = 5–6) at peak uptake and 60 min after injection, together with peak/60-min ratios for whole brain, cortex, hippocampus, striatum, and thalamus in wild-type (WT) mice. * denotes significant differences between [^18^F]F-DPA peak/60-min ratios and [^18^F]DPA-714 and [^11^C]PBR28 peak/60-min ratios. # denotes significant differences between [^18^F]DPA-714 peak/60-min ratios and [^11^C]PBR28 peak/60-min ratios. *p < 0.05, ** *p* < 0.01, *** *p* < 0.001, ^#^*p* < 0.05

**Table 2.** Averaged standardized uptake value ratios to cerebellum (SUVR_CB_) from different brain regions of transgenic APP/PS1-21 (TG) and wild-type (WT) mice at different periods after [^18^F]F-DPA, [^18^F]DPA-714, and [^11^C]PBR28 injection

		[^18^F]F-DPA			[^18^F]DPA-714			[^11^C]PBR28		
		TG	WT	TG/WT ratio	TG	WT	TG/WT ratio	TG	WT	TG/WT ratio
10–20 min	Brain	1.05 ± 0.03	0.84 ± 0.03	1.25	0.95 ± 0.02	0.92 ± 0.05	1.03 ^***^	0.97 ± 0.03	0.90 ± 0.03	1.08 ^***^
	Cortex	1.02 ± 0.05	0.70 ± 0.02	1.46	0.83 ± 0.03	0.79 ± 0.04	1.05 ^***^	0.86 ± 0.04	0.77 ± 0.03	1.12 ^***^
	Hippocampus	1.10 ± 0.06	0.74 ± 0.04	1.49	0.96 ± 0.04	0.92 ± 0.07	1.04 ^*** #^	1.01 ± 0.04	0.89 ± 0.06	1.13 ^***^
	Striatum	1.04 ± 0.05	0.67 ± 0.04	1.55	0.89 ± 0.02	0.82 ± 0.06	1.09 ^***^	0.96 ± 0.05	0.82 ± 0.04	1.17 ^***^
	Thalamus	0.97 ± 0.05	0.69 ± 0.06	1.41	0.98 ± 0.03	0.92 ± 0.08	1.07 ^*** #^	1.03 ± 0.05	0.90 ± 0.07	1.14 ^***^
20–40 min	Brain	1.06 ± 0.05	0.85 ± 0.04	1.25	0.95 ± 0.02	0.88 ± 0.05	1.08 ^***^	0.99 ± 0.03	0.87 ± 0.03	1.14 ^*^
	Cortex	1.03 ± 0.05	0.71 ± 0.03	1.45	0.86 ± 0.04	0.76 ± 0.04	1.13 ^*^	0.91 ± 0.04	0.74 ± 0.03	1.23
	Hippocampus	1.10 ± 0.07	0.73 ± 0.06	1.51	0.97 ± 0.04	0.86 ± 0.07	1.13^*** ##^	1.05 ± 0.05	0.83 ± 0.05	1.27^***^
	Striatum	1.03 ± 0.06	0.66 ± 0.05	1.56	0.91 ± 0.02	0.76 ± 0.05	1.19 ^***^	1.00 ± 0.05	0.77 ± 0.04	1.30 ^***^
	Thalamus	0.97 ± 0.05	0.66 ± 0.06	1.47	0.96 ± 0.03	0.82 ± 0.07	1.17 ^*** #^	1.02 ± 0.04	0.81 ± 0.07	1.26 ^***^
40–60 min	Brain	1.07 ± 0.05	0.87 ± 0.04	1.23	0.97 ± 0.02	0.86 ± 0.04	1.12 ^*^	1.02 ± 0.03	0.86 ± 0.03	1.19
	Cortex	1.05 ± 0.05	0.74 ± 0.03	1.42	0.88 ± 0.04	0.73 ± 0.04	1.21	0.96 ± 0.04	0.73 ± 0.03	1.31
	Hippocampus	1.09 ± 0.07	0.72 ± 0.04	1.51	0.98 ± 0.04	0.81 ± 0.06	1.21 ^*** ##^	1.08 ± 0.04	0.79 ± 0.04	1.37 ^*^
	Striatum	1.05 ± 0.07	0.68 ± 0.06	1.54	0.91 ± 0.03	0.71 ± 0.05	1.28 ^**^	1.04 ± 0.02	0.73 ± 0.04	1.42
	Thalamus	0.97 ± 0.05	0.67 ± 0.07	1.45	0.93 ± 0.03	0.74 ± 0.06	1.26 ^**^	1.03 ± 0.05	0.76 ± 0.06	1.36

Averaged SUVR_CB_ and standard deviations in whole brain, cortex, hippocampus, striatum, and thalamus of transgenic APP/PS1-21 (TG) and wild-type (WT) mice at different periods after injection (10–20, 20–40, and 40–60 min) using [^18^F]F-DPA, [^18^F]DPA-714, and [^11^C]PBR28 (*n* = 5–6). * denotes significant differences between [^18^F]F-DPA SUVR_CB_ TG/WT ratios, and [^18^F]DPA-714, and [^11^C]PBR28 SUVR_CB_ TG/WT ratios. # denotes significant differences between [^18^F]DPA-714 and [^11^C]PBR28 SUVR_CB_ TG/WT ratios. *p < 0.05, ** *p* < 0.01, *** *p* < 0.001, ^#^*p* < 0.05, ^##^*p* < 0.01

The residuals followed a normal distribution. Differences were considered significant for values of *p* < 0.05.

## Results

In previous studies we have performed longitudinal PET studies using [^18^F]F-DPA and [^18^F]DPA-714 in the APP-PS1/21 mouse model, together with parallel immunohistochemical studies to assess amyloid deposition and glial activation [11, 25]. Based on the previous studies, we chose to use 9-month-old mice in the current comparison due the extensive pathology in the AD model mice and clear PET signal.

### In vivo* [*^*18*^*F]F-DPA, [*^*18*^*F]DPA-714, and [*^*11*^*C]PBR28 Uptake in WT Mice*

**Fig. 1:**
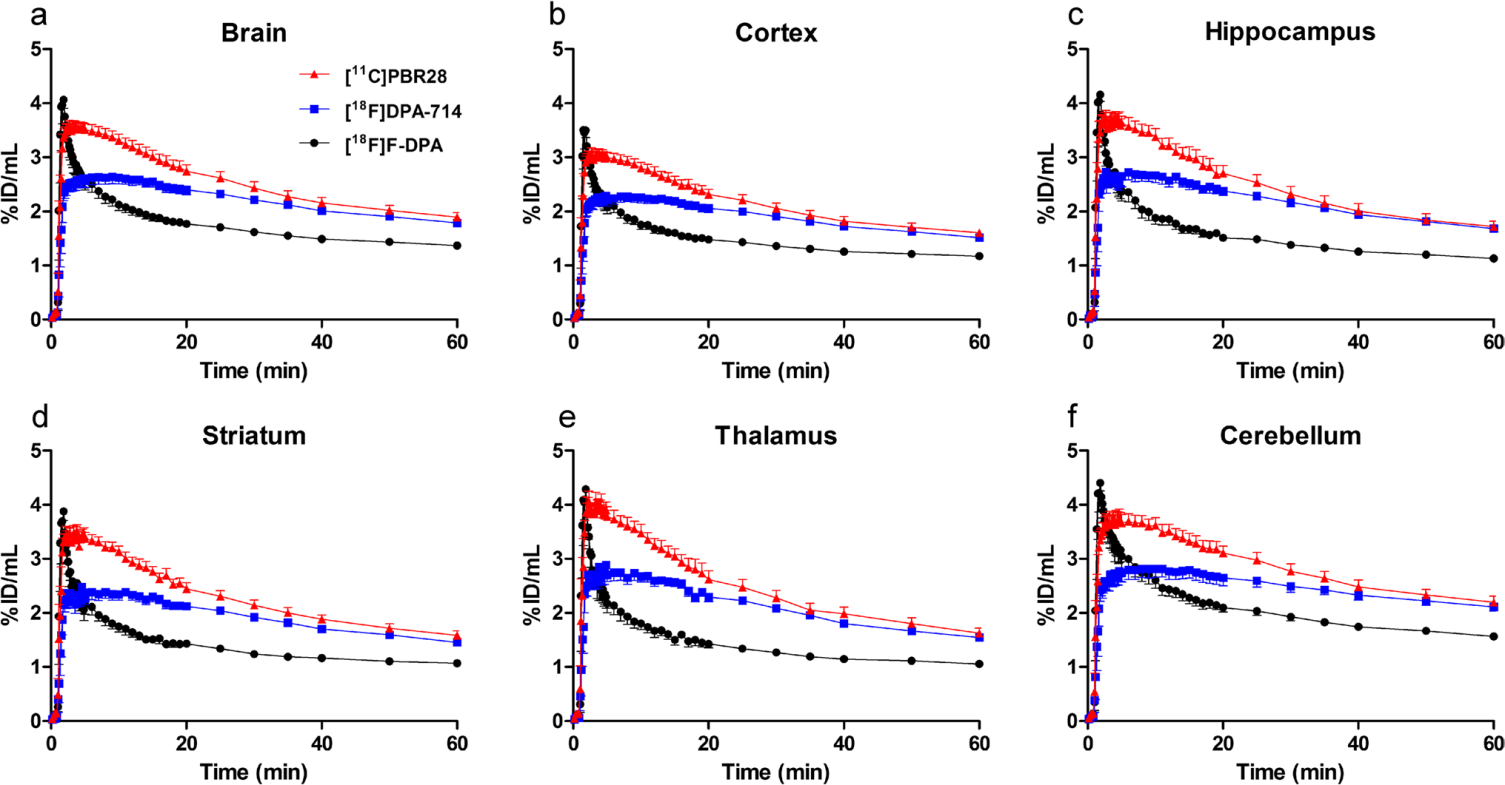
Time-activity curves as percentage of injected dose/mL (%ID/mL) for [^18^F]F-DPA, [^18^F]DPA-714, and [^11^C]PBR28 in whole brain (**a**), cortex (**b**), hippocampus (**c**), striatum (**d**), thalamus (**e**), and cerebellum (**f**) in the same wild-type (WT) mice (*n* = 6). Values are expressed as the average ± SD

The graphs in Fig. 1 show the 60-min time-activity curves for whole brain, cortex, HIPPO, STR, THA, and CB in WT mice. The results show the higher initial brain uptake and faster washout of [^18^F]F-DPA compared with [^18^F]DPA-714 and [^11^C]PBR28. Table 1 shows detailed uptake information as %ID/mL at peak uptake and 60 min after injection for [^18^F]F-DPA, [^18^F]DPA-714, and [^11^C]PBR28 for whole brain, cortex, HIPPO, STR, and THA in WT mice. The calculated peak/60-min ratios of [^18^F]F-DPA of all the studied regions were significantly higher (*p* < 0.001) than those of [^18^F]DPA-714 and [^11^C]PBR28. In the whole brain, the peak uptake values of [^18^F]F-DPA, [^18^F]DPA-714, and [^11^C]PBR28 were reached 2 min, 8 min, and 4 min, respectively, after the tracer injection.

### In vivo* [*^*18*^*F]F-DPA, [*^*18*^*F]DPA-714, and [*^*11*^*C]PBR28 Uptake in APP/PS1-21 Mice*

**Fig. 2:**
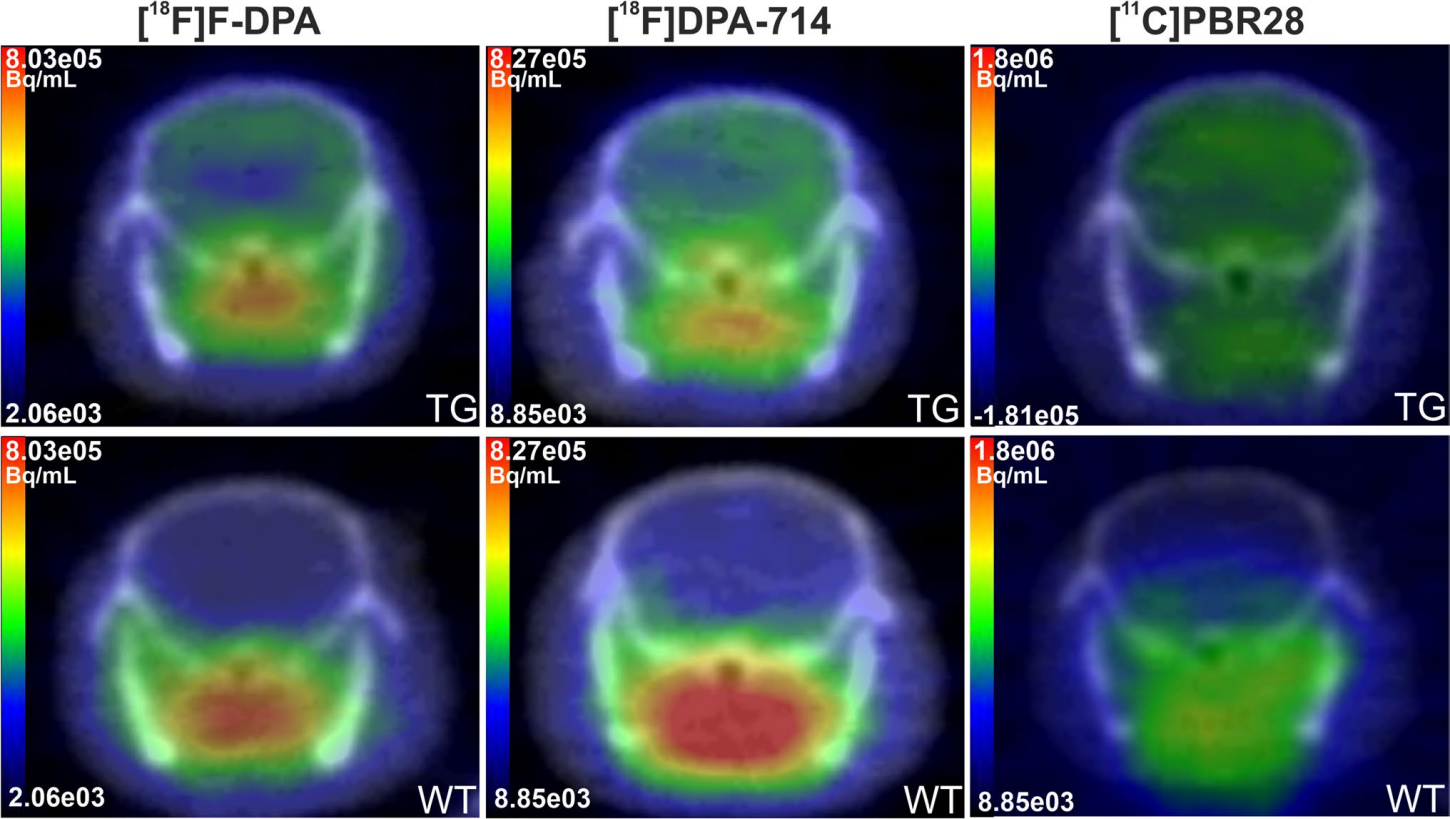
Representative 40–60-min summed images for [^18^F]F-DPA (left column), [^18^F]DPA-714 (middle column), and [^11^C]PBR28 (right column) in transgenic APP/PS1/21 (TG) (top row) and wild-type (WT) (bottom row) mice

**Fig. 3:**
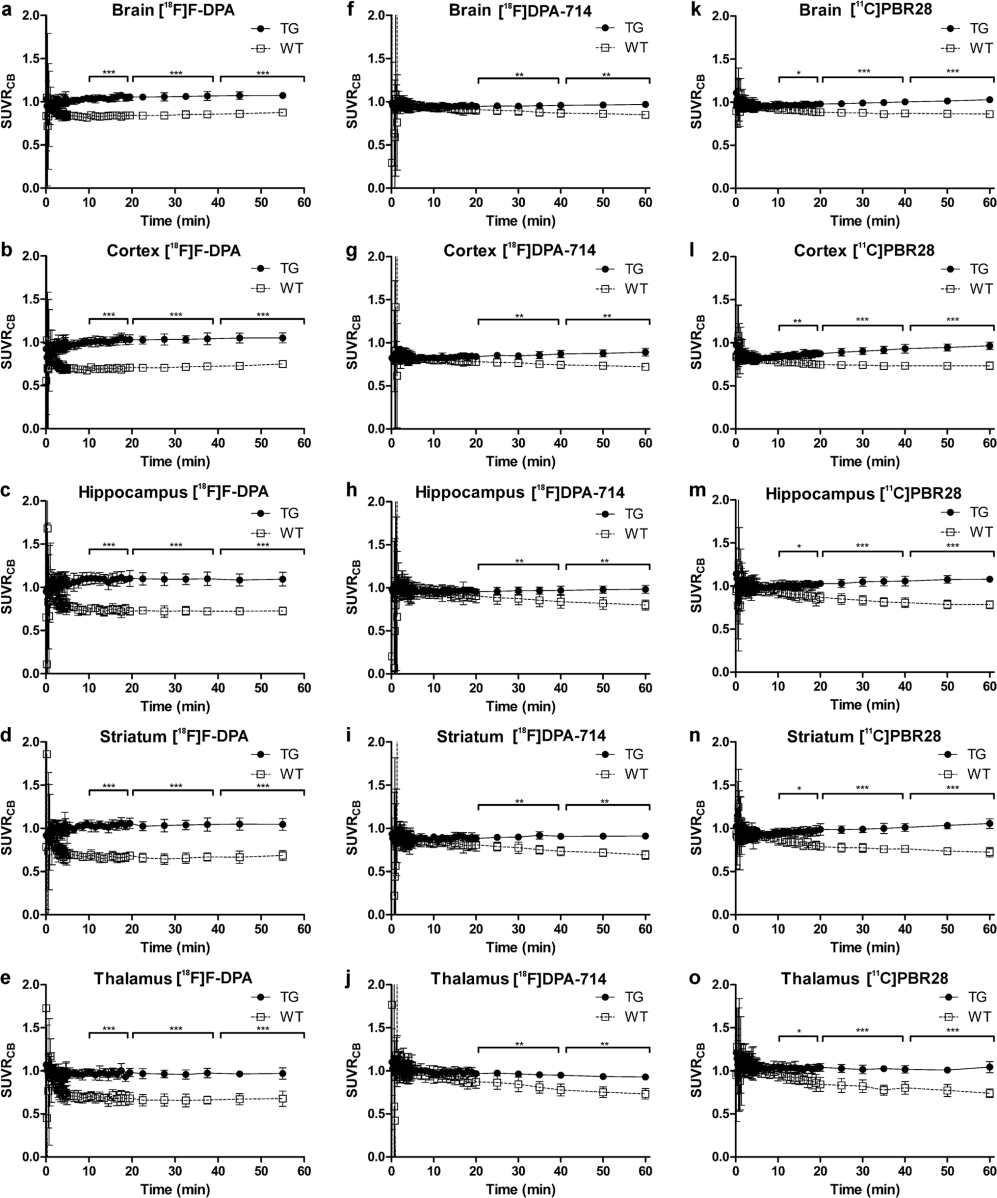
Standardized uptake value ratios to cerebellum (SUVR_CB_) differences between wild-type (WT) and transgenic APP/PS1/21 (TG) mice at different periods after injection (10–20, 20–40, and 40–60 min) in whole brain, cortex, hippocampus, striatum, and thalamus are shown for [^18^F]F-DPA (a-e), [^18^F]DPA-714 (f-j), and [^11^C]PBR28 (k–o). Values are expressed as the mean ± SD. *p < 0.05, ** *p* < 0.01, *** *p* < 0.001. The same WT (*n* = 6) and TG mice (*n* = 5–6) were imaged with all tracers

Together with the WT mice, 6 aged-matched APP-PS1/21 TG mice were scanned with [^18^F]F-DPA, [^18^F]DPA-714, and [^11^C]PBR28. Figure 2 shows representative images for [^18^F]F-DPA, [^18^F]DPA-714, and [^11^C]PBR28 in TG and WT mice. To compare [^18^F]F-DPA, [^18^F]DPA-714, and [^11^C]PBR28 in a mouse model of moderate neuroinflammation, we used 9-month-old APP/PS1-21 AD model mice. SUVR_CB_ values were calculated for the whole brain, cortex, HIPPO, STR, and THA in WT and TG mice, and the differences in SUVR_CB_ between WT and TG animals were calculated at 10–20, 20–40, and 40–60 min after radiotracer injection (Fig. 3).

For [^18^F]F-DPA, the SUVR_CB_ differences between WT and TG mice were highly significant (*p* < 0.001) in all the studied areas in the three studied periods (Figs. 3a–e). In the case of [^18^F]DPA-714, there were no significant differences in the 10–20-min time period, but the differences were significant in the 20–40-min (*p* < 0.01) and 40–60-min (*p* < 0.01) periods for all regions (Figs. 3f–j). For [^11^C]PBR28 at 10–20 min, the SUVR_CB_ differences between WT and TG were already significant for all the analyzed regions (*p* < 0.05), increasing in significance at 20–40 min (*p* < 0.001) and at 40–60 min (*p* < 0.001) (Figs. 3k–o).

Table 2 summarizes the averaged SUVR_CB_ values for TG and WT mice and the ratios of SUVR_CB_ values for TG and WT in the different time periods (10–20, 20–40, and 40–60 min) and regions for all three radiotracers. At the 10–20 min period, the [^18^F]F-DPA TG/WT ratios for all the studied brain regions were significantly higher (*p* < 0.001) than those of [^18^F]DPA-714 and [^11^C]PBR28. In addition, during that period, the TG/WT ratios of [^18^F]DPA-714 and [^11^C]PBR28 in the HIPPO and THA were also significantly different (*p* < 0.05).

In the 20–40-min period, high significance (*p* < 0.001) was still observed in the differences between the TG/WT ratios of [^18^F]F-DPA and [^18^F]DPA-714 in the whole brain, HIPPO, STR, and THA and between the TG/WT ratios of [^18^F]F-DPA and [^11^C]PBR28 in HIPPO, STR, and THA.

In the 40–60-min period, the differences between the TG/WT ratios of [^18^F]F-DPA and [^18^F]DPA-714 in HIPPO (*p* < 0.001), STR, and THA (*p* < 0.01) remained highly significant; however only low significance was seen between the TG/WT ratios of [^18^F]F-DPA and [^11^C]PBR28 in the HIPPO (*p* < 0.05). Significant differences were also detected for the HIPPO in all three time periods when comparing the TG/WT ratios of [^18^F]DPA-714 and [^11^C]PBR28.

**Fig. 4:**
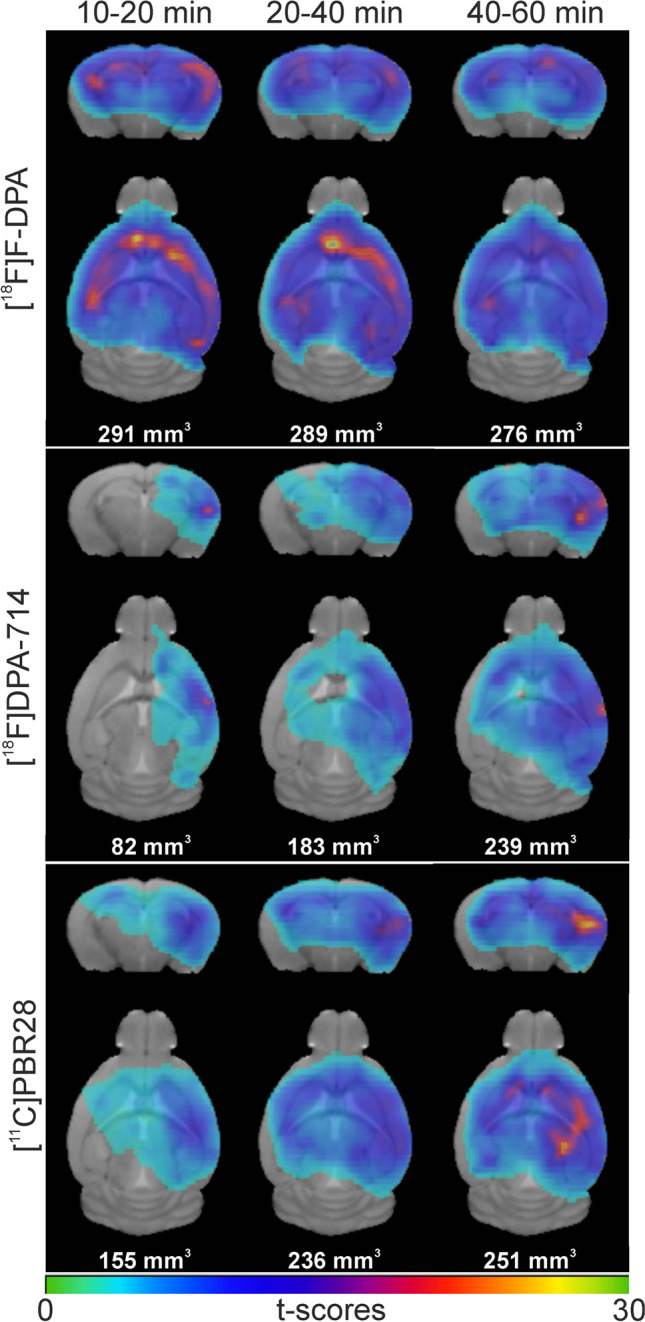
Voxel-wise two-sample t-test images and cluster sizes comparison of [^18^F]F-DPA (top), [^18^F]DPA-714 (middle), and [^11^C]PBR28 (bottom) brain uptake at different periods after injection (10–20, 20–40, and 40–60 min)

We performed a voxel-wise analysis of the PET images to study the differences in tracer uptake between WT and TG mice at 10–20, 20–40, and 40–60 min after injection at the voxel level. Figure 4 shows coronal and axial images of [^18^F]F-DPA, [^18^F]DPA-714, and [^11^C]PBR28 at the different time points and the cluster sizes. Due to the extensive signal, and after filtering, only a single cluster was detected. In the 10–20 min time frame, the cluster size for [^18^F]F-DPA was 292 mm^3^, while for [^18^F]DPA-714 and [^11^C]PBR28, the sizes were 83 mm^3^ and 156 mm^3^, respectively. In the next time frames, the [^18^F]DPA-714 and [^11^C]PBR28 cluster sizes increased significantly until the 40–60 min time frame. At this last time frame, the cluster sizes were 277 mm^3^ ([^18^F]F-DPA), 240 mm^3^ ([^18^F]DPA-714), and 251 mm^3^ ([^11^C]PBR28). Cortical and hippocampal uptake areas of higher significance are indicated by the higher t-scores. The voxel-based analysis also showed no differences in uptake between WT and TG in the CB.

## Discussion

Several studies have been performed in different animal models of AD using first-generation TSPO radiotracers such as [^18^F]FE-DAA1106 [26, 27], [^11^C]AC-5216 [27], and [^11^C]PK-11195 [4, 28], with marked disparities in the results. More recent studies using novel TSPO tracers such as [^11^C]PBR28 [29], [^18^F]GE-180 [30, 31], and [^18^F]DPA-714 [15, 16] have shown more consistent results in detecting inflammation.

In humans, increased microglial activation has been shown in *post mortem* brain samples of AD patients, although the role of this activation is still controversial [32, 33]. The first human PET studies targeting neuroinflammation were performed using [^11^C]PK11195, with contradictory results [34–37]. The varied findings with [^11^C]PK11195 could be explained by the variability in the studied populations, as well as the limitations of the radiotracer itself, including high non-specific binding, high lipophilicity, low blood–brain barrier penetration, and low binding potential [6, 7, 38]. Studies using newer TSPO radiotracers have also shown heterogeneous results in different populations [19, 39–41]. Among these novel tracers, [^18^F]DPA-714 has a better signal-to-noise ratio and a greater affinity than [^11^C]PK11195 [13]. The first human studies with [^18^F]DPA-714 concluded that this tracer cannot be used to distinguish individual AD patients from healthy subjects [19]. In contrast, a more recent human prospective study looked into early and protective microglial activation in AD and concluded that [^18^F]DPA-714 can be a good tool for assessing neuroinflammation in early and preclinical AD [20].

In the current study, we have shown in WT mice that [^18^F]F-DPA has better brain penetration and faster washout than [^18^F]DPA-714 and [^11^C]PBR28, as shown in Table 1 by the peak tracer uptake and the highly significant differences in peak uptake/60-min ratios. In addition, we compared the uptake in the brains of 9-month-old APP/PS1-21 mice. We have shown in two longitudinal studies that significant [^18^F]F-DPA and [^18^F]DPA-714 uptake can be measured in 9-month-old APP/PS1-21 model mice [11, 16] and therefore chose animals of that age for the direct tracer comparison. In the current study, higher SUVR_CB_ values were achieved with [^18^F]F-DPA compared with [^18^F]DPA-714 or [^11^C]PBR28, with significant differences between TG and WT mice in [^18^F]F-DPA uptake as soon as 20 min after the injection. In addition, the voxel-wise analysis confirmed the differences between the tracers, showing that the high uptake and fast washout of [^18^F]F-DPA allows the detection of differences in uptake between the WT and TG mice at earlier time frames compared with [^18^F]DPA-714 and [^11^C]PBR28. Interestingly the voxel-wise analysis showed an asymmetry for [^18^F]DPA-714 and for [^11^C]PBR28 in particular in the earlier time frames (10–20 and 20–40 min) compared with [^18^F]F-DPA in the same time frames; these asymmetries are attenuated in the last time frame (40–60 min), when the cluster sizes are also more similar. Given that the same animals were scanned with the three radiotracers, the most likely explanation is that this is due to the differences in uptake and washout speeds between the three radiotracers. This is in agreement with the results observed in Fig. 3, where in the last time frame (40–60 min) the smallest differences in SUVR_CB_ between WT and TG mice were observed for the three radiotracers.

The observed uptake differences between the closely related radiotracers [^18^F]F-DPA and [^18^F]DPA-714 could be due to the higher *in vivo* metabolic stability of [^18^F]F-DPA reported in rats and mice [11, 12]. In those studies, we showed that [^18^F]F-DPA is more metabolically stable than [^18^F]DPA-714 in the brain; non-metabolized [^18^F]F-DPA accounted for more than 90% of the remaining radioactivity even 90 min after injection, whereas for [^18^F]DPA-714, only about 50% of the brain activity was the parent compound. This can be explained by the direct ^18^F-labeling of the aromatic ring in [^18^F]F-DPA imparting a higher stability than the metabolically unstable alkoxy-linked ^18^F-label on the aromatic ring of [^18^F]DPA-714. Although the injected mass of [^18^F]F-DPA labeled by electrophilic ^18^F-fluorination was over 50-fold higher than the injected mass of [^18^F]DPA-714 in this study, [^18^F]F-DPA was better than [^18^F]DPA-714 for differentiating between TG and WT animals. Recently we have demonstrated that a 100-fold difference in injected mass of [^18^F]F-DPA in the same AD mouse model affected both the tracer kinetics and the tracer uptake. A higher injected mass resulted in a faster washout, with more rapid establishment of tracer equilibrium, but only an approximately 30% lower specific uptake [10]. [^18^F]DPA-714 and other TSPO binding radiotracers such as [^11^C]PK11195, [^11^C]DPA-713, [^18^F]GE-180, [^18^F]Fluoromethyl-PBR28, and [^18^F]CB251 have been used for head-to-head comparisons in ischemic stroke or experimental autoimmune myocarditis [42–45].

In this study, we used the CB as the reference region for analyses of the PET images. The use of the CB as a reference region is well established for amyloid quantification with [^11^C]PIB, but the choice of the CB is more controversial for analyzing the binding of TSPO tracers. We previously observed an age-dependent increase in tracer accumulation in the cerebellum; however, this increase resulted in a significant difference between TG and WT animals only from the age of 12 months onwards, with no significant difference observed at 9 months. The reference region (hypothalamus) employed in the previous study [10] is unsuitable as an *in vivo* reference region due to its proximity to the pituitary gland. Our use of the CB as the reference region for [^18^F]F-DPA, [^18^F]DPA-714, and [^11^C]PBR28 was based on our PET imaging data showing no differences in tracer uptake between TG and WT mice, as shown by the voxel-wise analysis in Fig. 4, and the cerebellar time-activity curves in Suppl. Figure 1. In addition, in previous studies, the CB has proven to be a reliable reference region, and its use as such also decreases group variability [11, 16, 46]. From our study we can conclude that the novel TSPO radiotracer [^18^F]F-DPA shows higher initial brain uptake, faster clearance, and better target-to-background ratios than [^18^F]DPA-714 and [^11^C]PBR28 when the comparisons are made with the same AD and WT animals. Furthermore, due to the washout kinetics, higher SUVR_CB_ values are measured with [^18^F]F-DPA and at earlier time points after injection. With all of these characteristics, the novel tracer [^18^F]F-DPA could prove very useful for the detection of low levels of microglial activation inflammation and, because of its fast clearance, would permit shorter dynamic scans.

## Supplementary Information

Below is the link to the electronic supplementary material.

Supplementary Fig. S1 (PNG 178 KB)

Supplementary file1 (TIF 57 KB)

## Data Availability

All data were handled in accordance with the comprehensive quality system in effect at the Turku PET Centre and following the open science and research data policy in use by the University of Turku (https://www.utu.fi/sites/default/files/public%3A//media/file/data-policy.pdf). The raw data from the PET animal imaging studies and laboratory analyses are archived in the Turku PET Centre archiving systems. These data will be available upon agreement with the Turku PET Centre.
