# Supplementary figures and images for: Direct Comparison of [18F]F-DPA with [18F]DPA-714 and [11C]PBR28 for Neuroinflammation Imaging in the same Alzheimer’s Disease Model Mice and Healthy Controls
Source: Mol Imaging Biol. 2021 Sep 20;24(1):157–66. doi: 10.1007/s11307-021-01646-5 (PMC8760190; doi:10.1007/s11307-021-01646-5)

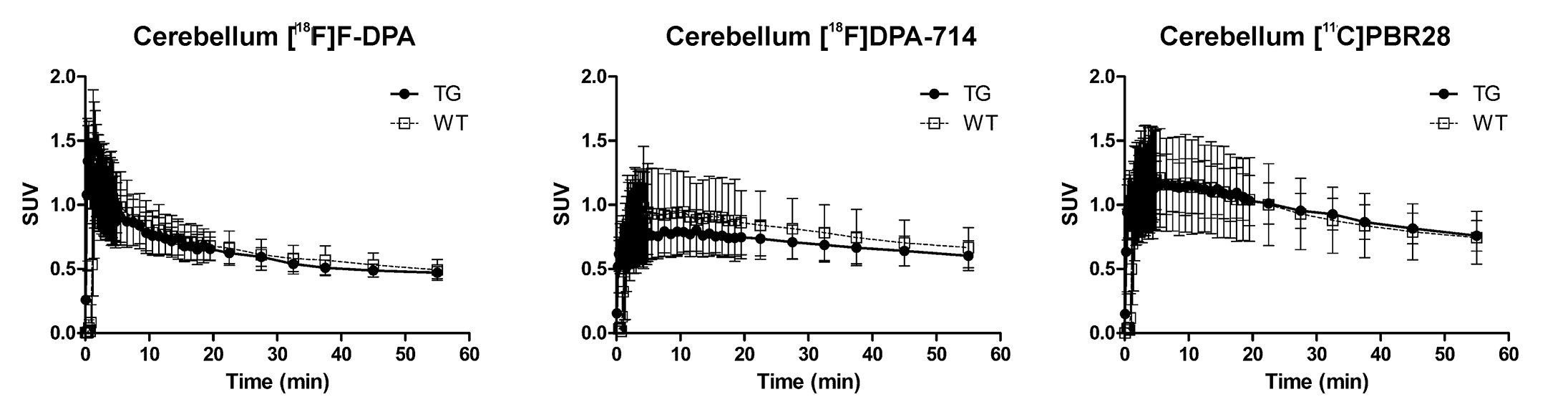

Supplement: Supplementary file 1 — (PNG 178 KB) [file 11307_2021_1646_Fig5_ESM.png]
